# O-GlcNAcylation in health and neurodegenerative diseases

**DOI:** 10.1038/s12276-021-00709-5

**Published:** 2021-11-26

**Authors:** Byeong Eun Lee, Pann-Ghill Suh, Jae-Ick Kim

**Affiliations:** 1 Department of Biological Sciences, Ulsan National Institute of Science and Technology (UNIST), Ulsan, 44919 Republic of Korea; 2 Korea Brain Research Institute (KBRI), Daegu, 41062 Republic of Korea

**Keywords:** Neurodegeneration, Glycobiology, Alzheimer's disease, Parkinson's disease

## Abstract

O-GlcNAcylation is a posttranslational modification that adds O-linked β-*N*-acetylglucosamine (O-GlcNAc) to serine or threonine residues of many proteins. This protein modification interacts with key cellular pathways involved in transcription, translation, and proteostasis. Although ubiquitous throughout the body, O-GlcNAc is particularly abundant in the brain, and various proteins commonly found at synapses are O-GlcNAcylated. Recent studies have demonstrated that the modulation of O-GlcNAc in the brain alters synaptic and neuronal functions. Furthermore, altered brain O-GlcNAcylation is associated with either the etiology or pathology of numerous neurodegenerative diseases, while the manipulation of O-GlcNAc exerts neuroprotective effects against these diseases. Although the detailed molecular mechanisms underlying the functional roles of O-GlcNAcylation in the brain remain unclear, O-GlcNAcylation is critical for regulating diverse neural functions, and its levels change during normal and pathological aging. In this review, we will highlight the functional importance of O-GlcNAcylation in the brain and neurodegenerative diseases.

## Neurodegeneration: Sugar tags on proteins linked to brain disease

The addition of a sugar tag called O-GlcNAc to proteins found in brain cells plays a critical role in regulating synaptic and neuronal function, with implications for understanding and treating neurodegenerative disease. Jae-Ick Kim and colleagues, Ulsan National Institute of Science and Technology, South Korea, review the ways in which the attachment or removal of O-GlcNAc sugars to or from proteins in the central nervous system can impact neuronal survival and the functional properties of neural circuits. In the aging brain, O-GlcNAc levels often become dysregulated, leading to aberrant protein activity that can fuel cognitive decline. In various neurodegenerative disorders such as Alzheimer’s, Parkinson’s, and Huntington’s diseases, and amyotrophic lateral sclerosis, relevant disease-associated proteins often have abnormal O-GlcNAc patterns. Therapeutically altering the sugar-tagging process could potentially offer a new way of treating these brain disorders.

## Introduction

**Fig. 1:**
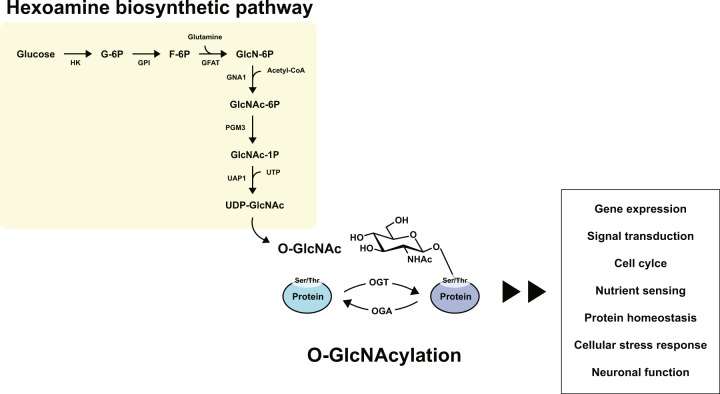
O-GlcNAcylation and its regulation of various cellular processes. O-GlcNAcylation is a posttranslational modification that attaches O-GlcNAc moieties to serine or threonine residues of cellular proteins. O-GlcNAcylation can regulate important cellular processes such as gene expression, signal transduction, cell cycle, nutrient sensing, protein homeostasis, cellular stress response, and neuronal function.

O-GlcNAcylation, which attaches O-linked β-*N*-acetylglucosamine (O-GlcNAc) moieties to either serine or threonine residues of intracellular proteins, is a posttranslational modification that critically regulates essential cellular functions^1–7^. Notably, this modification is catalyzed by only two enzymes, O-GlcNAc transferase (OGT) and O-GlcNAcase (OGA). OGT catalyzes the addition of O-GlcNAc to the hydroxyl groups of serine/threonine residues of nucleocytoplasmic proteins, whereas OGA removes the modification from proteins (Fig. 1)^1,3,4,8,9^. O-GlcNAcylation occurs in various cellular locations, such as the nucleus, cytosol, and cellular organelles, including mitochondria, the cytoskeleton, and the endoplasmic reticulum^10^. O-GlcNAcylation by OGT utilizes uridine diphosphate *N*-acetylglucosamine (UDP-GlcNAc) as a precursor molecule, which is synthesized by the hexosamine biosynthesis pathway (HBP) that is pivotal for the cellular metabolism of amino acids, lipids, and nucleotides in the cell (Fig. 1)^1^. In the HBP, glucose is first phosphorylated by hexokinase (HK) and converted to glucose-6-phosphate (G-6P). Next, G-6P is transformed into fructose-6-phosphate (F-6P) by phosphoglucose isomerase (GPI). Then, glutamine:fructose-6-phosphate aminotransferase (GFAT), a rate-limiting enzyme of HBP, catalyzes F-6P and glutamine into glucosamine-6-phosphate (GlcN-6P). With acetyl-CoA, GlcN-6P is further catalyzed by glucosamine-phosphate *N*-acetyltransferase (GNA1) and turns into *N*-acetylglucosamine-6-phosphate (GlcNAc-6P). Further isomerization by GlcNAc phosphomutase (PGM3) produces *N*-acetylglucosamine-1-phosphate (GlcNAc-1P). Finally, with uridine triphosphate (UTP), UDP-GlcNAc, and pyrophosphate (PPi) are produced by UDP-GlcNAc pyrophosphorylase (UAP1) (Fig. 1)^2,11^. Notably, since O-GlcNAcylation occurs on serine/threonine residues of proteins, the O-GlcNAcylation site can also be phosphorylated, and there could be extensive crosstalk between O-GlcNAcylation and phosphorylation^3,8,12^. Hence, it is conceivable that O-GlcNAcylation, through its competitive interplay with phosphorylation, could critically affect a variety of cellular signaling pathways by dynamically modulating protein activities^8,13^.

Mounting evidence has shown that O-GlcNAcylation regulates essential cellular processes such as gene expression, signal transduction, cell cycle, nutrient sensing, protein homeostasis, and cellular responses to diverse stress conditions (Fig. 1)^13–18^. In addition, altered and abnormal O-GlcNAcylation is frequently observed in multiple disease conditions, including cancer, cardiac hypertrophy, and type II diabetes^19–23^, supporting the notion that O-GlcNAcylation is implicated not only in maintaining normal cellular functions but also in the pathological processes of human diseases. Notably, among bodily organs, O-GlcNAcylation is the most abundant in the brain^10^. Furthermore, two related enzymes, OGT and OGA, show high expression and activity in the brain^24,25^. These findings strengthen the emerging notion that O-GlcNAcylation plays an important role in various neural functions. Therefore, in this review, we will focus on the importance of O-GlcNAcylation in neural functions and neurodegenerative diseases.

## O-GlcNAcylation in the brain

**Fig. 2:**
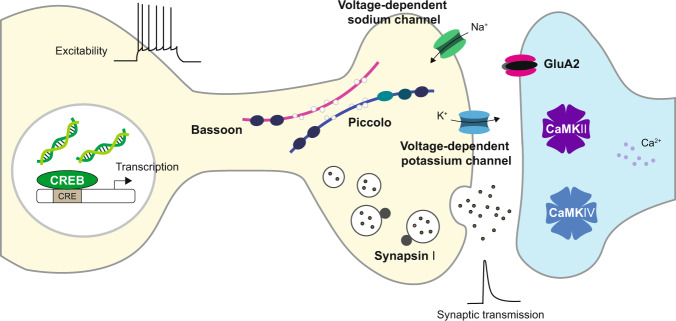
Neuronal and synaptic proteins modified by O-GlcNAcylation. Various neuronal and synaptic proteins, including bassoon, piccolo, synapsin I, GluA2, CaMKII, CaMKIV, and CREB, are modified by O-GlcNAcylation. These modifications critically regulate neuronal and synaptic properties.

The physiological importance of O-GlcNAcylation in the brain is reflected by the fact that both OGT and OGA are indispensable for neuronal survival. Genetic deletion of OGT caused embryonic stem cell lethality, while deletion of OGA led to perinatal lethality^26–28^. In addition, although it is still not clear how these changes occur, neuron-specific elimination of either OGT or OGA results in severe structural abnormalities in the nervous system, which seems to be caused either by neurodevelopmental defects or premature neurodegeneration^29–32^. More importantly, recent studies are beginning to show that O-GlcNAcylation is crucial not only for neuronal survival but also for neuronal and synaptic function in the mature brain. O-GlcNAcylation in the brain is highly enriched, especially at synapses. OGA and OGT are significantly active at functional synapses, leading to extensive O-GlcNAc modification of proteins in nerve terminals^33^. Moreover, many synaptic and neuronal proteins important for structure and function, such as bassoon, synapsin I, piccolo, synaptopodin, GluA2, calcium/calmodulin-dependent kinases II (CaMKII), CaMKIV, and cyclic adenosine monophosphate (AMP)-response element-binding protein (CREB), are modified by O-GlcNAcylation (Fig. 2)^15,34–38^. O-GlcNAcylation of these proteins was found to critically regulate the functional properties of neurons in multiple neural circuits.

When O-GlcNAc was acutely increased by an OGA inhibitor, thiamet-G (TMG), neuronal excitability, and excitatory synaptic transmission of hippocampal neurons were suppressed. A transient enhancement of O-GlcNAcylation elevated the amplitude of the voltage-dependent potassium channel current and the expression of hyperpolarization-activated cyclic nucleotide-gated (HCN) channels and reduced the size of voltage-gated sodium channels^39^. This suppression of neuronal excitability by O-GlcNAcylation in multiple cation channels clearly demonstrates the ability of O-GlcNAc modification to regulate neuronal function. Agouti-related protein (AgRP)-expressing neurons in the hypothalamus showed enriched OGT expression, and selective knockout of OGT in these AgRP neurons inhibited their spontaneous firing activity due to the defective action of the voltage-gated potassium channel Kcnq3 (Kv7.3) by loss of O-GlcNAc modification in this potassium channel^40^. Forebrain excitatory neuron-specific deletion of OGT by using mice expressing a tamoxifen-inducible Cre recombinase under the control of the CaMKIIα promoter also promoted hyperphagia-dependent obesity by attenuating excitatory input to the paraventricular nucleus (PVN) neurons in the hypothalamus^41^. Consistent with this finding, OGT is enriched in the postsynaptic density of excitatory synapses, and removal of OGT from cultured cortical neurons markedly reduced the number of mature excitatory synapses by regulating the synaptic expression of α-amino-3-hydroxy-5-methyl-4-isoxazolepropionic acid (AMPA) receptors^42^. Dopaminergic neuron-specific elimination of OGT caused early loss of axonal arborization and premature neurodegeneration, whereas enhancement of O-GlcNAcylation by knocking out OGA in dopamine neurons did not negatively affect neuronal structures. Interestingly, these dopamine neuron-specific OGA knockout mice exhibited enhanced dopamine release at dopamine terminals in the striatum, suggesting the potential role of O-GlcNAcylation in modulating the synaptic release machinery^32^. These studies collectively indicate that neuronal O-GlcNAcylation significantly regulates not only the survival or maintenance of neurons but also the physiological functions of synapses and neurons in the brain. Given the diverse roles played by O-GlcNAcylation in key cellular processes, much needs to be identified with respect to O-GlcNAcylated neuronal proteins, their O-GlcNAcylation sites, and the brain-specific signaling mechanisms selectively affected by O-GlcNAc modification.

## O-GlcNAcylation and neurodegenerative diseases

### Aging

Considering the critical role of O-GlcNAcylation in regulating synaptic and neuronal functions, it is reasonable to assume that the alteration of O-GlcNAc levels during aging could exert deleterious effects on numerous neural functions in the brain. Several reports have shown that the abundance of O-GlcNAcylation changes depending on aging conditions^43–48^. In the hippocampus of aged mice, both OGT expression and the level of O-GlcNAcylation were decreased compared with those in young mice, and this loss of O-GlcNAcylation by age impaired cognitive function^44^. Age-dependent loss of O-GlcNAcylation in neural stem cells (NCS) is correlated with reduced neurogenesis and increased gliogenesis in the hippocampus^47^. Notably, decreased O-GlcNAcylation in the NSCs of young mice by conditional knockout of OGT led to a reduction in adult neurogenesis and impaired hippocampal-dependent learning and memory^47^. The relationship between O-GlcNAcylation and aging was also found in invertebrate organisms such as *Caenorhabditis elegans*. The *oga-1 C. elegans* mutant showed altered gene expression compared with the wild-type, and these differentially expressed genes were largely associated with the aging pathway and lifespan^49^. Moreover, modulating O-GlcNAc levels by manipulating HBP revealed a protective effect on the aging and lifespan of *C. elegans*. Lifespan was significantly increased in the *gfat-1* gain-of-function mutants, and supplementation with GlcNAc was sufficient to extend the lifespan by alleviating proteotoxicity and improving protein quality control^50^. Supplementation with glucosamine (GlcN) also resulted in an extended lifespan in both nematodes and aged mice^51^. Collectively, these findings identify a critical role of O-GlcNAcylation and related pathways in aging and lifespan.

Moreover, according to recent studies, alterations of O-GlcNAcylation in various neuronal proteins are found in aging-related neurodegenerative diseases, including Alzheimer’s disease (AD) and Parkinson’s disease (PD)^52,53^. Numerous proteins associated with neurogenerative diseases appear to be O-GlcNAcylated, and their O-GlcNAcylation status can change the progression of disease pathology (Table 1)^8,54,55^. Tau protein, one of the best-known hallmarks of AD, is O-GlcNAcylated in the brain^56–58^. It has been shown that the O-GlcNAcylation of tau protein mitigates pathological aggregates of tau and, as a consequence, ameliorates cellular toxicity caused by aggregated tau^59^. Thus, elucidation of the alterations of O-GlcNAcylation in neurodegenerative diseases not only provides a more comprehensive view of the role of O-GlcNAcylation in the brain but also is vital to the identification of novel therapeutic targets and therapies against currently untreatable neurodegenerative diseases such as AD.

**Table 1:** O-GlcNAcylation status in aging and neurodegenerative diseases.

Condition	Species	Brain region (cell type)/molecular target	O-GlcNAc status	Phenotype	References
Aging	Mouse	Whole-brain	Decreased	-	^48^
		Hippocampus	Decreased	Impaired cognitive function	^44^
		Hippocampus (neural stem cell)	Decreased	Impaired neurogenesis, gliogenesis, and hippocampal-dependent learning	^47^
		Whole-body	Increased (by GlcN supplementation)	Extended lifespan	^51^
	*C. elegans*	Whole-body	Increased (by *gfat-1* gain-of-function mutation or supplementation of GlcNAc)	Extended lifespan	^50^
			Increased (by GlcN supplementation)		^51^
Alzheimer’s disease (animal model)	Human	Frontal cortex, Brodmann area 7, inferior parietal lobule	Decreased	-	^57,61–63^
	Mouse	Brain, cervical spinal cord/tau	Increased (by pharmacological OGA inhibition)	Reduced pathological tau phosphorylation, attenuated neurofibrillary tangles, and neuronal death	^59^
		Amyloid-beta (Aβ)	Increased (by pharmacological OGA inhibition or genetic upregulation of OGA expression)	Reduction of Aβ by attenuated γ-secretase activity, reduction of activated microglia and astrocyte, reduced neuronal death, recovery of impaired memory function	^63,69^
	Rat	Hippocampus/tau	Increased (by pharmacological OGA inhibition)	Reduced tau phosphorylation	^66^
	Cultured cells	PC-12 cells	Increased (by pharmacological OGA inhibition)	Reduced tau phosphorylation	^66^
Huntington’s disease (animal model)	Mouse	Cortex/nucleoporin (NUP)	Decreased	Mislocalized NUPs	^80^
	Cultured cells	Primary cortical neurons/huntingtin	Increased (by pharmacological OGA inhibition)	Reduced cell death	^80^
		Neuro2A cells	Decreased (by genetic OGA expression)	Reduced mutant huntingtin aggregation and cell death	^76^
Amyotrophic lateral sclerosis (animal model)	Mouse	Ventral horn of the spinal cord	Decreased	Reduced number of motor neurons	^85^
		Spinal cord	Decreased	Excessive ROS, motor neuron death	^91^
	Rat	Spinal cord/neurofilament	Decreased	Neurofilament loss	^89^
	Cultured cells	SH-SY5Y cell/TDP-43	Increased (by genetic upregulation of OGT expression or by GlcNAc treatment)	Attenuated aggregation of abnormal TDP-43 and cellular toxicity	^94^
Parkinson’s disease (animal model)	Mouse	α-synuclein	Increased (by genetic and pharmacological enhancement of O-GlcNAc)	Reduced α-synuclein aggregation, reduced dopaminergic neuron death, recovered dopamine release, and motor function	^32^
	Cultured cells	SH-SY5Y cell, hippocampal neuron, SK-N-SH neuroblastoma cells/α-synuclein	Increased (by site-specific mutation or pharmacological upregulation)	Reduced α-synuclein aggregation, reduced cell death, less toxicity, reduced α-synuclein preformed fibril (PFF) uptake	^106,107,109^

### Alzheimer’s disease

Alzheimer’s disease (AD) is the most common neurodegenerative disease and is characterized by progressive mental deterioration. Symptoms start with mild memory deficits and mood changes and progress to severe cognitive impairment and difficulties in swallowing and urination, eventually resulting in death^60^. Recently, researchers found that the O-GlcNAc level is 22 to 50% lower in AD patients than in healthy controls^57,61–63^. As such, it is plausible that O-GlcNAcylation is associated with the pathogenesis and/or progression of AD pathology. In addition to amyloid-beta, tau protein has been shown to play a major role in the pathology of AD^64,65^. Tau is a microtubule-associated protein that modulates the stability of the neuronal cytoskeleton, and the activity of tau protein, like many other proteins, is highly regulated by phosphorylation. In the AD brain, tau is abnormally hyperphosphorylated and then accumulates. This accumulation of hyperphosphorylated tau eventually forms neurofibrillary tangles (NFTs) that induce significant cellular toxicity^64,65^. Importantly, tau protein is extensively O-GlcNAcylated^56,57^, and several studies have revealed that O-GlcNAcylation of tau can effectively reduce tauopathy. Treatment with an OGA inhibitor, thiamet-G, in PC-12 cells expressing tau protein substantially diminished tau phosphorylation at Ser396 and Thr231, the initial priming phosphorylation sites of pathological tau, and this result was also replicated in the rat brain after thiamet-G treatment in vivo^66^. Among AD mouse models, the JNPL3 mouse is a transgenic mouse model that overexpresses mutant human P301L tau. This mouse develops neurofibrillary tangles, gliosis, and neurodegeneration in an age-dependent manner due to tau hyperphosphorylation^67^. When the JNPL3 mice were treated with thiamet-G, the phosphorylated tau at Ser202 and Thr205 was remarkably reduced in the brain. Moreover, this decrease in pathological tau phosphorylation led to the attenuation of NFT formation and neuronal death, suggesting a neuroprotective effect of O-GlcNAcylation in the AD brain^59^.

Amyloid-β (Aβ) plaques are another hallmark of AD. Aβ is a peptide whose toxic oligomeric form is widely found in the brains of AD patients^68^. The 5xFAD mouse is a well-studied AD model overexpressing human mutant Aβ precursor protein and shows severe amyloid pathology, including elevated extracellular Aβ plaques. When 5xFAD mice were treated with an OGA inhibitor, the number of Aβ plaques was remarkably reduced compared to that in vehicle-treated mice^69^. This reduction in Aβ plaques was caused by the attenuated activity of γ-secretase, a critical enzyme for Aβ generation. Furthermore, the activity of γ-secretase was attenuated through O-GlcNAcylation at the Ser708 residue of nicastrin (NCT), which is an essential component of the γ-secretase complex and acts as a substrate receptor^69,70^. From the above, it is apparent that in addition to the regulation of NFT formation, O-GlcNAcylation might be critical for the modulation of γ-secretase activity and the resulting Aβ production. The reactivity of microglia and astrocytes was also significantly reduced by OGA inhibition, and this reduction in amyloid pathology resulted in the recovery of impaired memory function in 5xFAD mice^69^. Genetically increased O-GlcNAc levels due to insufficient OGA also recovered impaired cognitive function in 5xFAD mice (5xFAD;OGA^fl/+^)^63^. The amount of Aβ in 5xFAD;OGA^fl/+^ mice was significantly reduced compared with that in 5xFAD mice, resulting in attenuation of neuronal death. This alleviation of pathology in 5xFAD;OGA^fl/+^ mice, in turn, markedly restored cognitive function^63^. Given that the elevation of O-GlcNAcylation does not cause any harmful effects on the structure and function of neurons^32,59^, it is conceivable that increasing O-GlcNAcylation would have a neuroprotective effect against AD with minimal side effects. Recently, selective and small-molecule OGA inhibitors such as MK-8719 and ASN120290 (previously known as ASN-561) have entered early clinical trials for the treatment of progressive supranuclear palsy (PSP)^71^. Moreover, LY3372689, another OGA inhibitor, is being developed for the treatment of tauopathies, including AD. Hopefully, the results from these clinical trials may be utilized to justify whether brain-permeable OGA inhibitors can be pursued for further clinical development against AD.

### Huntington’s disease

Huntington’s disease (HD) is a rare, inherited neurodegenerative disease that is characterized by progressive neurodegeneration, motor impairment, and cognitive dysfunction^72,73^. The main cause of HD is a mutation of the huntingtin (*HTT*) gene coding the huntingtin protein (HTT). Originally, the *HTT* gene contains up to 34 glutamine-coding (CAG) repeats. By mutations, this repeat expands to polyglutamine repeats and causes misfolding of the huntingtin protein^74^. This abnormally folded protein is prone to aggregate, readily forming oligomers. These oligomers are seeds for larger inclusions and pathogenic mutant huntingtin (mHTT) fibrils^74^. Functionally, these mHTT fibrils and aggregates interrupt synaptic transmission, mitochondrial axonal transport, and gene transcription^74^. To date, the relationship between O-GlcNAcylation and HD has been unclear. Interestingly, however, HTT protein and huntingtin-interacting protein 1-related protein (HIP1R) appear to be O-GlcNAc-modified, indicating that HTT protein could be functionally regulated by O-GlcNAcylation^32,75^. When the HTT protein construct with polyglutamine repeats was coexpressed with OGA in Neuro2A cells (murine neuroblastoma cell line), the number of transfected cells with mutant huntingtin aggregates was significantly reduced. Coexpression of the OGA constructs also led to a significant reduction in cell death mediated by mHTT aggregation^76^. In contrast to the findings in cases of AD, these results support the possibility that lowering O-GlcNAcylation levels can mitigate the aggregation and cellular toxicity of the mHTT protein.

In HD mouse models, distortion of the nuclear membrane and disrupted nucleocytoplasmic transport were also observed^77^. Nucleoporin (NUP) is a component of the nuclear pore complex that is required for selective transport across the nuclear pore. Notably, mislocalization of this NUP was found in HD mouse models. R6/2 and zQ175 mice are HD mouse models expressing human mHTT with expanded CAG repeats^78,79^. In R6/2 mice that develop progressive motor and cognitive deficits as early as 6-8 weeks of age, NUP62, which is important for controlling nuclear pore permeability and selective transport, formed intranuclear inclusions and was colocalized with mHTT aggregates^80^. Consistent with this finding, intracellular inclusions formed by NUP88 were found and colocalized with mHTT aggregates in zQ175 mice^80^. This NUP mislocalization and pathology were also observed in postmortem human brains from HD patients^80^. Intriguingly, NUP is heavily O-GlcNAcylated, and its O-GlcNAcylation appears to be critical for nuclear pore integrity and selective filtration^81^. Strengthening this result, there was a reduction in O-GlcNAcylation in zQ175 mice, and treatment of primary cortical neurons transfected with HTT 82Q with an OGA inhibitor markedly reduced cell death, indicating that elevation of O-GlcNAc levels could restore nucleocytoplasmic trafficking^80^. On the basis of these observations, NUP mislocalization in HD pathology is likely linked to altered levels of O-GlcNAcylation. Thus, it is increasingly evident that O-GlcNAcylation may be related to HD pathology by modulating mHTT aggregation and nucleocytoplasmic transport. Further elucidation of the exact role of O-GlcNAcylation in HD pathology will lead to a better understanding of the molecular etiology and pathophysiology of HD.

### Amyotrophic lateral sclerosis

Amyotrophic lateral sclerosis (ALS), also known as Lou Gehrig’s disease, is a neurodegenerative motor neuron disease that causes the degeneration of both lower and upper motor neurons in the motor cortex, brain stem nuclei, and anterior horn of the spinal cord^82–84^. This loss of motor neurons results in focal limbic muscle weakness and progresses to respiratory failure, limiting survival to 2–4 years after disease onset^82,83^. Nevertheless, the exact mechanism of motor neuron degeneration remains incompletely understood. Several factors, such as genetic mutation, abnormal neurofilament function, oxidative stress, and inflammation, are considered the main causes of ALS^84^. In ALS, 90% of cases are classified as sporadic ALS, and the remaining 10% of cases are familial ALS with dominantly inherited autosomal mutations in SOD1 (superoxide dismutase 1), TDP-43 (TAR DNA-binding protein 43), FUS (fused in sarcoma/translated in liposarcoma), and C9orf72 (chromosome 9 open reading frame 72)^83^. According to recent studies of ALS, O-GlcNAcylation can play a neuroprotective role against ALS pathology. It was reported that the abundance of O-GlcNAcylation in mutant SOD1-overexpressing mice was significantly reduced in the motor neurons of the spinal cord compared with that of wild-type mice^85^. Interestingly, hyperphosphorylated neurofilaments (NFs) and their aggregation are observed in ALS pathology^86,87^, and this neurofilament is modified by O-GlcNAc^32,88^. Consistent with these findings, O-GlcNAcylation of neurofilaments remarkably decreased in the ALS rat model overexpressing mutant SOD1, potentially indicating the disruption of O-GlcNAcylation in ALS pathology^89^. Considering the crosstalk with phosphorylation, O-GlcNAcylation may suppress excessive phosphorylation of neurofilaments and concomitantly alleviate ALS pathology.

In addition, the elevation of oxidative stress is one of the primary factors leading to the degeneration of motor neurons in ALS^83,84^. Nonselenocysteine-containing phospholipid hydroperoxide glutathione peroxidase (NPGPx) is an oxidative stress sensor and transmitter that modulates protein activity by shuffling disulfide bonds^90^. Notably, NPGPx knockout mice showed ALS-like phenotypes such as paralysis, denervation of neuromuscular junctions, and motor neuron loss. Importantly, these mice displayed dysregulation of O-GlcNAc levels and ROS accumulation, consequently causing motor neuron death. However, this loss of motor neurons was recovered by elevating O-GlcNAc levels with an OGA inhibitor^91^. These results suggest that O-GlcNAcylation can modulate ALS pathology by reducing ROS accumulation. TDP-43 protein pathology is also a hallmark of ALS. TDP-43 is an RNA/DNA-binding protein and acts as a regulator of transcription, mRNA stability, and transport^92^. The excessive accumulation of TDP-43 in the cytoplasm produces inclusion bodies, which induce cellular toxicity by abnormal protein/RNA interactions^84,93^. A recent study reported that the O-GlcNAcylation of endogenous TDP-43 was found in human SH-SY5Y neuroblastoma cells, and mass spectrometry analysis further detected O-GlcNAcylation at the T199 and T233 sites of TDP-43^94^. As in the aforementioned cases, coexpression of OGT with an ALS-linked mutant of TDP-43 significantly suppressed abnormal TDP-43 aggregation and related cellular toxicity^94^. Together, these studies indicate a critical role of O-GlcNAcylation in modulating ALS pathology by inhibiting excessive phosphorylation and protein aggregation and reducing ROS accumulation. The molecular mechanisms underlying the role of O-GlcNAcylation in ALS pathology need to be further elucidated.

### Parkinson’s disease

Parkinson’s disease (PD) is the second most common neurodegenerative disease after AD. PD patients generally develop motor symptoms, including rigidity, bradykinesia, and postural instability^95–98^. However, alongside these motor symptoms, they also experience nonmotor symptoms such as sleep disruption, depression, and even cognitive decline years before the diagnosis of PD^98,99^. The loss of dopaminergic neurons in the midbrain and the functional disruption of the basal ganglia circuitry are the key features of PD pathophysiology^96,100^. Among pathological markers of the disease, Lewy bodies (LBs), composed of an abnormal aggregation of proteins, are a well-known pathological hallmark of PD that can induce neuronal toxicity, and α-synuclein is the major component of LB inclusions^101,102^. α-Synuclein, contributing a large proportion of PD pathology, is expressed abundantly in the brain and is primarily located in presynaptic terminals and synaptic vesicles^102,103^. Although the function of α-synuclein is still unclear, α-synuclein is known to regulate the mobility, release, and maintenance of synaptic vesicles^102,103^. As with other aggregation-prone proteins, α-synuclein phosphorylation can induce abnormal aggregation, and this aggregation, in turn, is capable of significantly disturbing cellular structure and function, which eventually causes neuronal death^104^. Notably, α-synuclein is also O-GlcNAc modified^104,105^. When α-synuclein was O-GlcNAcylated in a site-specific manner, the abnormal aggregation of α-synuclein by phosphorylation was markedly diminished, resulting in alleviated neurotoxicity in cultured neurons^106–108^. In these experiments, α-synuclein containing O-GlcNAc at T72 did not form any aggregates or oligomers and inhibited phosphorylation at S87 and S129, which has been found to be closely related to α-synuclein aggregation in PD pathology. In addition, treatment with O-GlcNAcylated α-synuclein in cultured neurons reduced neuronal death^106^. Despite its variable impact depending on the specific O-GlcNAcylation sites, O-GlcNAcylated α-synuclein largely showed an inhibitory effect on aggregation. The O-GlcNAcylation of α-synuclein at T72, T75, and T81 remarkably inhibited or attenuated α-synuclein aggregation compared to that of unmodified α-synuclein, and triple O-GlcNAcylation of α-synuclein at all of these sites completely blocked aggregation^107^. In a similar vein, the elevation of O-GlcNAc in neuroblastoma cells by OGA inhibition reduced the uptake of α-synuclein preformed fibrils (PFFs) without interrupting the normal endocytosis capacity^109^. Taken together, these findings strongly indicate that O-GlcNAcylation of α-synuclein can attenuate pathological aggregation of α-synuclein and α-synuclein-mediated neurotoxicity in vitro.

Recent findings from our group also demonstrated that O-GlcNAcylation plays an important role in the function, survival, and degeneration of dopamine neurons in vivo in a mouse model of PD^32^. In our study, dopaminergic neuron-specific OGT knockout mice exhibited a significant loss of dopamine neurons in the midbrain and premature death at ~8 to 15 weeks of age. In contrast, selective enhancement of O-GlcNAcylation in dopamine neurons did not negatively impact neuronal structures or survival. Rather, it facilitated synaptic transmission at dopamine synapses in mice with OGA conditional knockout^32^. These results indicate that O-GlcNAcylation is essential for neuronal survival and function in dopamine neurons. More interestingly, when we generated a mouse model of PD by overexpressing mutant α-synuclein in the midbrain of mice with OGA conditional knockout, the elevated O-GlcNAcylation dramatically alleviated PD pathology in dopamine neurons, including abnormal aggregation of α-synuclein and neuronal death. As a functional consequence of these changes, impaired dopamine release and motor behaviors observed in PD model mice were significantly recovered by enhanced O-GlcNAcylation^32^. Together, the elevation of O-GlcNAc levels in vivo can alleviate PD pathology and related physiological symptoms, possibly by inhibiting the pathological aggregation of α-synuclein. Given that both genetic and pharmacological elevation of O-GlcNAc did not cause any harmful effects on brain function and general health in mice^32^, it may be clinically promising to develop PD therapies focusing on O-GlcNAcylation. More research should be conducted to identify molecular targets of O-GlcNAcylation in dopamine neurons and their pathophysiological roles in PD.

## Conclusion and perspectives

**Fig. 3:**
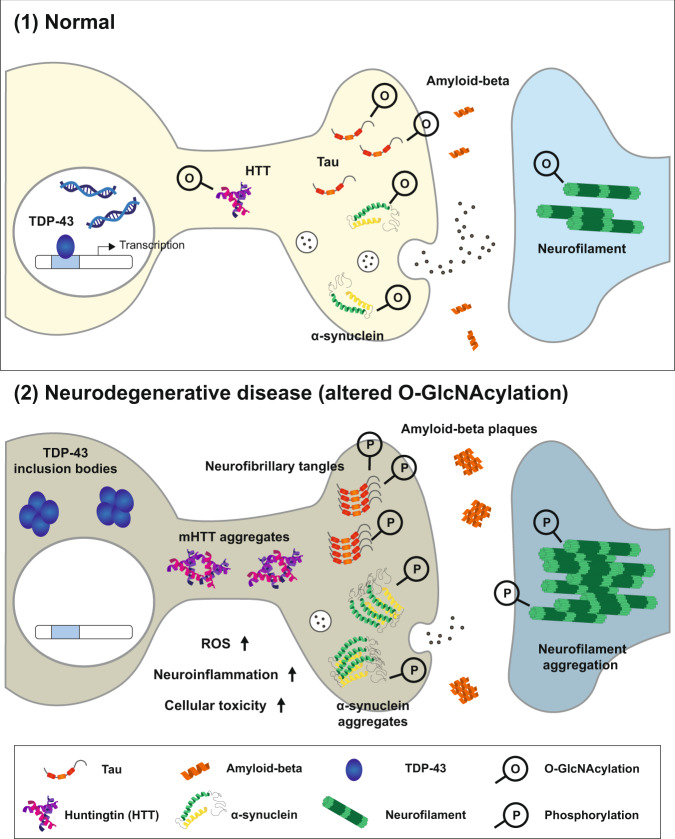
O-GlcNAcylation and its neuroprotective role in neurons. Key etiological proteins of neurodegenerative diseases, such as tau, α-synuclein, HTT, and neurofilament, can be directly O-GlcNAcylated, and proteins, including amyloid-beta and TDP-43, are indirectly affected by O-GlcNAcylation. In neurodegenerative diseases, altered O-GlcNAcylation is detected, and this abnormal O-GlcNAc status and subsequent excessive phosphorylation can cause pathological protein aggregation, resulting in cellular toxicity in neurons.

O-GlcNAcylation critically contributes to various cellular processes, including transcription, translation, signaling cascades, and protein homeostasis, in a multitude of cell types, and more than 5000 human proteins have been identified as O-GlcNAcylated proteins thus far^10^. Notably, recent studies on O-GlcNAcylation have begun to emphasize the importance of O-GlcNAcylation in the central nervous system. There is undoubtedly evidence indicating a pivotal role of O-GlcNAcylation in the brain. First, O-GlcNAc modification is most abundant in the brain among various organs. Second, numerous proteins enriched in and important for functional synapses are O-GlcNAcylated. Most importantly, accumulating studies have demonstrated that genetic or pharmacological manipulation of O-GlcNAcylation remarkably alters neuronal and synaptic functions in the brain. Moreover, the critical role of O-GlcNAcylation is not limited to the normal, healthy brain. Several proteins known as key etiological factors in neurodegenerative diseases, including tau, amyloid-beta precursor protein (APP), α-synuclein, and HTT, are O-GlcNAcylated. In addition, the status of O-GlcNAcylation in these proteins is associated with many pathological conditions in neurodegenerative diseases. In experimental studies, altering O-GlcNAc levels in the brain or cultured neurons reduces aberrant protein aggregation, which is a common hallmark of neurodegenerative diseases. Moreover, the manipulation of O-GlcNAcylation (mostly enhancement) in neurodegenerative disease-related proteins mitigates neurotoxicity, functional impairment, and neuronal death, strongly suggesting the neuroprotective effect of O-GlcNAcylation (Fig. 3). It is important to note that, in most cases, either genetic or pharmacological manipulation of O-GlcNAc levels does not lead to any deleterious effects on the structure and function of neurons in the brain; instead, it specifically suppresses neurodegenerative disease-related pathology in animal models. Thus, it is worth pursuing O-GlcNAcylation as a therapeutic target for many neurodegenerative diseases, such as AD and PD.

However, caution should be exercised because O-GlcNAcylation is a dynamic modification that reversibly interacts with other posttranslational modifications, including phosphorylation, ubiquitylation, acetylation, and methylation, and causes complicated modulation of diverse proteins in the cytoplasm and nucleus^110^. Depending on the specific brain regions, cell types, and pathological conditions, the effect of O-GlcNAc modification in the brain can be variable and may even be harmful in some cases. In addition, another important issue researchers should address when investigating O-GlcNAcylation is the reliable detection of O-GlcNAcylated proteins, which is still challenging for several reasons. First, since the donor substrate of O-GlcNAc modification, UDP-GlcNAc, is produced by the sequential integration of nutrients, including glucose, amino acids, fatty acids, and nucleotides, via HBP, O-GlcNAcylation is highly sensitive to the metabolic states of cells in living animals^2,3,12^. Second, even though O-GlcNAcylation itself is abundant in the brain, each individual O-GlcNAc-modified protein is generally present in low abundance^33^. Finally, O-GlcNAcylation is labile and easily lost from peptides during the process of dissociation^111^. Fortunately, however, recent progress in proteomics and new emerging strategies in the detection of O-GlcNAcylation have enabled us to overcome these aforementioned difficulties/issues^112^. In addition, the development of single-cell isolation and analysis can further provide detailed profiling of individual cell-specific responses, ranging from gene expression to proteomics^113^. Undoubtedly, this technology may greatly encourage the interpretation of neuron type-specific and protein-specific effects of O-GlcNAcylation in the near future.

In this review, we briefly described recent progress demonstrating the neuroprotective effects of O-GlcNAcylation in a variety of neurodegenerative diseases. Our understanding of O-GlcNAcylation in the brain continues to evolve at a rapid pace, but its practical application for therapeutic goals is still in its infancy. Moving forward, the utilization of advanced new technologies will allow us to dissect the molecular alterations by O-GlcNAcylation that determine disease-specific pathology and its alleviation in neurodegenerative diseases.
